# Efficacy and safety of ruxolitinib in the treatment of chronic graft-versus-host disease: a retrospective analysis

**DOI:** 10.1007/s00277-024-05697-w

**Published:** 2024-06-25

**Authors:** Alexander Denk, Cornelia Mittermaier, Daniela Weber, Matthias Fante, Sibel Güneş, Matthias Edinger, Wolfgang Herr, Daniel Wolff

**Affiliations:** 1 Department of Internal Medicine IIIHematology and Oncology, University Hospital Regensburg, Franz-Josef-Strauss-Allee 11, 93053 Regensburg, Germany; 2 Novartis Pharma AG, Basel, Switzerland

**Keywords:** Chronic graft versus host disease, Allogeneic hematopoietic stem cell transplantation, Ruxolitinib, Steroid-refractory chronic graft-versus-host disease, JAK 1/2 inhibitor

## Abstract

Steroid-refractory chronic graft-versus-host disease (cGvHD) is associated with significant morbidity and mortality, with ruxolitinib being the first drug approved for its treatment. We retrospectively analyzed the safety and efficacy of ruxolitinib for treatment of cGvHD at our center between 07/2015 and 12/2022 and identified 48 patients receiving ruxolitinib as second (18/48) or advanced (30/48) treatment line. Ruxolitinib was started on median day 340 (range 119–595) after cGvHD onset; median duration of administration was 176 (range, 79–294) days with 16/48 patients continuing treatment at last follow-up. National Institutes of Health organ grading and the intensity of immunosuppression were assessed at the start of ruxolitinib treatment and repeated after 1, 3, 6, and 12 months. Response assessment was terminated at the start of any additional new immunosuppressant treatment. The median time of follow-up was 582 (range, 104–1161) days. At the primary analysis after six months on ruxolitinib treatment, the overall response rate was 33%, and failure-free survival was 58%. Infectious adverse events ≥ CTCAE grade III were observed in 10/48 patients. The response rate was not associated with the severity of cGvHD, number of previous treatment lines, or number of additional agents combined with ruxolitinib applying a univariate regression model. At the time of the 12-month follow-up, four patients experienced recurrence of the underlying malignancy and two patients had experienced non-relapse-related mortality. Overall, ruxolitinib was relatively well-tolerated and showed outcomes comparable to the REACH3 trial in a heavily pretreated patient population.

## Introduction

Chronic graft-versus-host disease (cGvHD) represents a major complication after allogeneic stem cell transplantation (allo-HSCT) that significantly contributes to non-relapse mortality (NRM) and reduced quality of life [1, 2]. According to current data, cGvHD occurs in approximately 30–50% of patients, as recent modifications in GvHD prophylaxis and regimens and host compatibility contributed to a decline in its prevalence [3–5]. As a result of chronic inflammation and impaired tolerance mechanisms resulting in host-reactive immune response [6, 7], onset is usually observed between 3 months and 2 years after allo-HSCT [8]. Standard first-line treatment for cGvHD is glucocorticosteroids (GS) with or without calcineurin inhibitors [9, 10]. Unfortunately, approximately 30–50% of patients lack response to first-line treatment, which consecutively leads to an increased risk of morbidity and mortality in this population [11, 12].

Since the Janus kinase/signal transducers and activators of transcription (JAK/STAT) signaling pathway is crucial for the activation of immune cells and tissue inflammation during GvHD, ruxolitinib, an orally applied JAK 1/2 inhibitor, has been explored for the treatment of GvHD [13, 14]. Based on encouraging results in second-line treatment of steroid-refractory, acute GvHD (aGvHD), ruxolitinib was first approved in 2019 as a second-line therapy [15]. Subsequently, following the promising outcomes reported in the randomized controlled REACH3 trial, which compared ruxolitinib with other available therapies for treatment of cGvHD, ruxolitinib was approved by the United States Food and Drug Administration and the European Medicines Agency [16, 17]. Because the REACH3 trial was conducted in a select patient population evaluating ruxolitinib as second-line therapy, and real-world data are not yet sufficiently available, there is an urgent need for data from unselected patients in routine clinical practice, including those receiving advanced treatment lines. Therefore, we retrospectively analyzed the efficacy and safety of all patients receiving ruxolitinib for the treatment of cGvHD between 2015 and 2022 at the University Hospital Regensburg in Germany.

## Patients and methods

### Patients

48 patients who received ruxolitinib for the treatment of cGvHD between July 2015 and December 2022 at the University Hospital Regensburg (Germany) were included in this retrospective analysis, which was approved by the institutional review board of the University of Regensburg (no. 22-3076-104). The analysis was conducted in accordance with the current Declaration of Helsinki. Diagnosis, assessment of organ involvement, and documentation of cGvHD were conducted as part of routine clinical practice using the National Institutes of Health consensus criteria [10] either during inpatient therapy or at outpatient follow-up visits.

### Definition of response to ruxolitinib treatment and adverse events

Clinical response was evaluated at 1 month, 3 months, 6 months, and 12 months after the start of ruxolitinib therapy. If a new immunosuppressive medication (ISM) was given after the start of ruxolitinib treatment, the response assessment was discontinued. Complete remission (CR) was defined as the resolution of all symptoms of cGvHD without initiation of new or additional ISM during treatment with ruxolitinib. Partial remission (PR) was defined as an improvement of at least one organ grade without progression of cGvHD in other organs, whereas mixed response (MR) was defined as an improvement in one organ, while progression occurred elsewhere. Progressive disease (PD) was defined as progression of at least one organ site without any improvements in other sites. Stable organ involvement without any changes in grading was classified as stable disease (SD). For evaluation of predictive markers, patients were divided into “responder” (CR, PR) and “non-responder” (MR, SD, PD, and additional ISM) categories. Failure-free survival (FFS) was defined as absence of relapse of the underlying disease or NRM, and no addition of further ISM. Calculation of overall response rates (ORRs) was based on an intention-to-treat analysis. If a patient did not complete the entire follow-up period of 12 months, the respective patient was excluded from ORR and FFS calculations from the first follow-up time point that had not been completed (i.e., 1 month, 3 months, 6 months, or 12 months) onward. To assess infectious adverse events (AEs) and hematological toxicities, the Common Terminology Criteria for Adverse Events version 5.0 (CTCAE 5.0) was used.

### Statistical analyses

Statistical analyses were conducted with SPSS Statistics 26 (SPSS Inc, Chicago, Illinois). Absolute numbers, percentual frequency (n, %), and median including interquartile range (IQR) are shown. Due to the limited number of patients, only univariate analyses were conducted. The effect of ten clinical parameters on the response to ruxolitinib treatment were analyzed using univariate binary logistic regression; the parameters were the patient’s age, sex, ruxolitinib dose at onset of therapy, azole comedication, prednisolone dose at onset of therapy, severity of cGvHD, number of additional ISMs, prior therapy lines, CTCAE thrombocytopenia, and CTCAE anemia at the start of treatment. Assessment of the GS-sparing effect during ruxolitinib treatment was conducted by non-parametric matched pairs analysis (Wilcoxon signed rank test). Assessment of GS dosing/weaning was captured among all patients, regardless of response, as long as no other ISM was added. Comparison of cytopenia at the start of treatment and within 6 months after ruxolitinib treatment (paired nominal data) was conducted using nonparametric McNemar test; Assessment of severe AEs regarding cytopenia was also conducted using the nonparametric McNemar test. The level of significance was set at *p*_two−sided_ ≤0.050.

## Results

### Characteristics of patients

Patient characteristics and history of aGvHD are summarized in Table 1. The median age at the start of ruxolitinib treatment was 51 years (range, 42–58 years). Underlying malignancies were myeloid disorders in 31 patients, and lymphatic malignancies in 17 patients (Table 1). 41 patients had received peripheral blood stem cells and seven patients had received bone marrow as a graft source. Prior acute GvHD grade II or higher, according to Glucksberg criteria, was observed in 32 patients (67%).

**Table 1:** Detailed characteristics of the patients, including sex, age, diagnosis, donor type, stem cell source, GvHD prophylaxis, and history and onset of acute GvHD

Characteristics	cGvHD *n* = 48
Male, n (%)	29 (60)
Female, n (%)	19 (40)
Age at start of ruxolitinib treatment, median years (range)	51 (42–58)
*Diagnosis*	*n (%)*
AML	22 (46)
NHL	7 (15)
ALL	5 (10)
MPN	4 (8)
MDS	5 (10)
Multiple myeloma	3 (6)
Hodgkin lymphoma	2 (4)
*Donor type*	**n (%)**
HLA-matched unrelated	23 (48)
HLA-mismatched unrelated	2 (4)
HLA-matched related	18 (38)
Haploidentical related	5 (10)
GvHD after DLI	5 (10)
Female donor / male recipient (n, %)	8 (17)
*Stem cell source*	
Peripheral blood stem cells	41 (85)
Bone marrow	7 (15)
**GvHD prophylaxis**	
ATG/CyA/MTX	22 (46)
CyA/MTX	13 (27)
Cyclo/Tacro/MMF	7 (15)
ATG/CyA/MMF	2 (4)
CyA/MMF	2 (4)
Cyclo/Everolimus/MMF	1 (2)
Tacro/MMF	1 (2)
**History of aGvHD**	*n (%)*
Grade 0-I	16 (33)
Grade II-IV	32 (67)
aGVHD onset, median days after allo-HSCT (range)	19 (16–23)

Abbreviations: aGvHD = acute GvHD; AML = acute myeloid leukemia, ALL = acute lymphoblastic leukemia, ATG = anti-thymocyte globulin, cGvHD = chronic GvHD; CyA = cyclosporine A; Cyclo = cyclophosphamide; DLI = donor lymphocyte infusion; GvHD, graft-versus-host-disease; MDS = myelodysplastic syndrome; MMF = mycophenolate mofetil; MTX = methotrexate; MPN = myeloproliferative neoplasia; NHL = Non-Hodgkin lymphoma; RD = related; URD = unrelated; Tacro = tacrolimus

Seventeen patients (35%) developed overlap cGvHD, while 31 patients (65%) had classic cGvHD onset. At the start of ruxolitinib treatment, 20 patients (42%) experienced severe cGvHD, 22 patients (46%) moderate cGvHD, and six patients (13%) residual mild cGvHD.

The majority of the patients had steroid-refractory cGvHD (*n* = 36, 75%), while 12 patients had steroid-dependent cGvHD (25%). The most common manifestations of cGvHD were the skin (*n* = 32, 67%), eyes (*n* = 28, 58%), and oral mucosa (*n* = 24, 50%), while fascia and the lung were involved in 15 (31%) and in 11 patients (23%), respectively.

Onset of cGvHD was on median day 202 (range, 136–282). Ruxolitinib was started on median day 622 (range 402–899) after allo-HSCT and on median day 340 (range, 119–595) after onset of cGvHD with a median dose of 2 × 10 mg/day (range 10–20 mg/d).

Patients had received a median of two prior treatment lines (range, 1–3) for cGvHD. At onset of ruxolitinib treatment, 31 patients (65%) received one additional ISM, 14 patients (29%) two additional ISMs, two patients (4%) three additional ISMs, and one patient (2%) no other ISM. The median duration of ruxolitinib treatment was 176 days (range, 79–294), and the median follow-up period after last administration of ruxolitnib was 582 days (range 104–1161 days), with 16 patients having ongoing therapy at the date of last assessment. Treatment characteristics and concomitant ISM are provided in Table 2.

**Table 2:** Treatment characteristics and concomitant immunosuppressive medication

Characteristics	Value
Onset of cGvHD, median days after allo-HSCT (range)	202 (136–282)
Start of ruxolitinib after allo-HSCT, median days (range)	622 (402–899)
Start of ruxolitinib treatment after onset of cGvHD, median days (range)	340 (119–595)
Ruxolitinib dose (mg/day) at onset of therapy, median (range)	20 (10–20)
Duration of ruxolitinib application, median days (range)	176 (79–294)
Duration of follow up, median days (range)	582 (104–1161)
*cGvHD maximum severity before start of ruxolitinib*	*n (%)*
Mild	2 (4)
Moderate	23 (48)
Severe	23 (48)
*cGvHD severity at start of ruxolitinib*	
Mild	6 (13)
Moderate	22 (46)
Severe	20 (42)
*Type of cGvHD*	
Overlap	17 (35)
Classic	31 (65)
*Steroid response of cGvHD*	
Steroid resistant	36 (75)
Steroid dependent	12 (25)
*Number of organs involved by cGvHD at start of ruxolitinib*	
One	11 (23)
Two	12 (25)
Three	13 (27)
Four or more	12 (25)
*Type of cGvHD organ involvement*	
Skin	32 (67)
Oral	24 (50)
Eyes	28 (58)
Gut	5 (10)
Liver	7 (15)
Lung	11 (23)
Musculoskeletal	15 (31)
Genital	2 (4)
Polyserositis	1 (2)
Meningoencephalitis	1 (2)
Polyneuropathy	1 (2)
Kidney involvement	1 (2)
*ISM at the start of ruxolitinib*	
No ISM	1 (2)
One ISM	31 (65)
Two ISMs	14 (29)
Three ISMs	2 (4)
*Number of prior treatment lines before ruxolitinib*	
One	18 (38)
Two	15 (31)
Three	9 (19)
Four or more prior therapies	6 (13)

Abbreviations: allo-HSCT = allogeneic hematopoietic stem cell transplantation; allo-SCT, allogeneic stem cell transplantation; cGvHD, chronic GvHD, GvHD, graft-versus-host disease; ISM = immunosuppressive medication; IST, immunosuppressive therapy; IQR, interquartile range; med, median

Using univariate regression analyses, none of the following nine factors (patient’s age, sex, initial ruxolitinib and prednisolone dose, severity of cGvHD, number of additional ISMs, prior therapy lines, CTCAE thrombocytopenia, and CTCAE anemia at start of treatment) were significantly associated with treatment response at the 6-month follow-up.

### Response to ruxolitinib

#### Response to ruxolitinib at 1 month

**Fig. 1:**
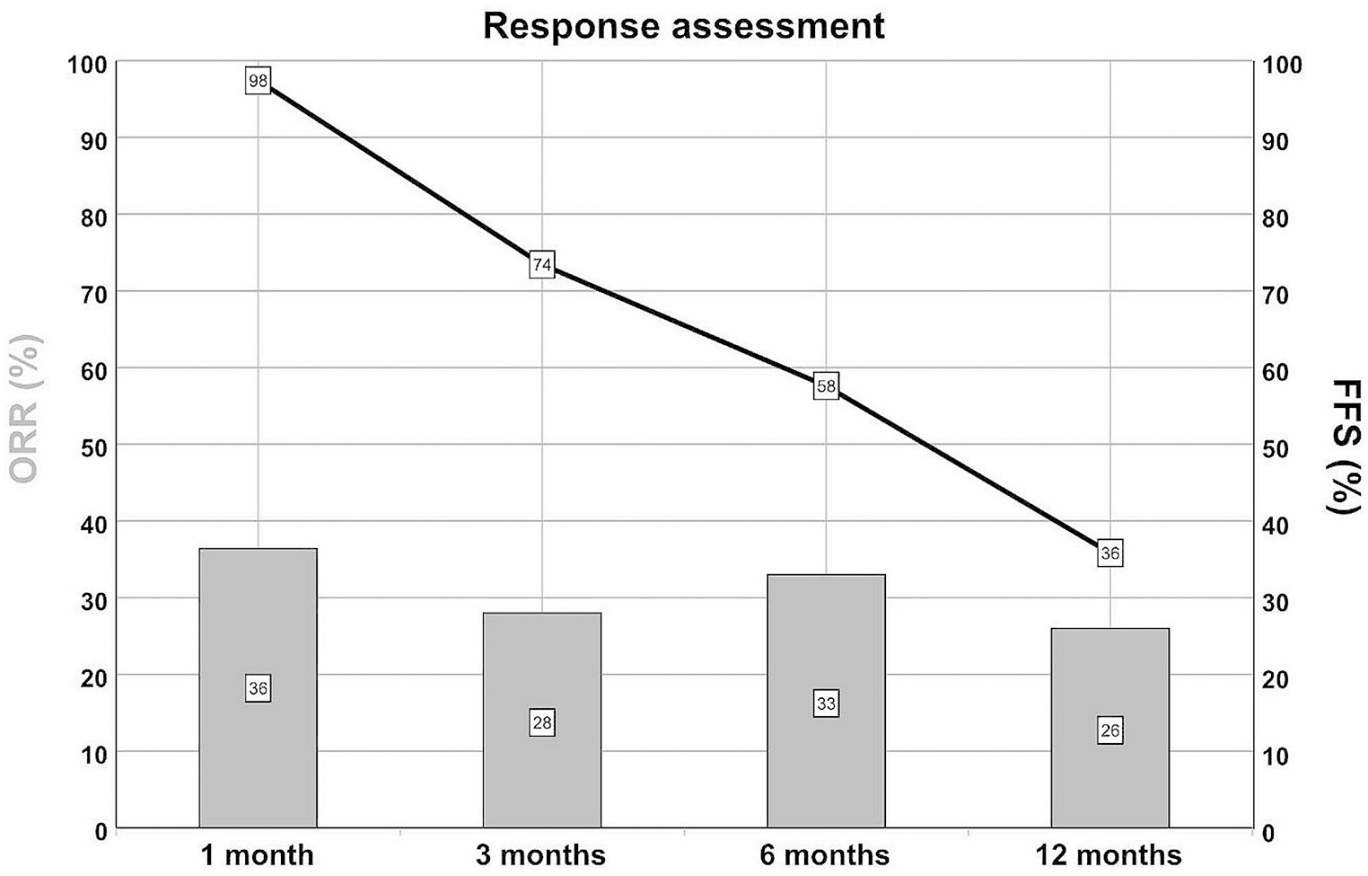
Response assessment. Overall response rates (ORR, columns) and failure-free survival (FFS, line) over time after initiation of ruxolitinib therapy: ORR and FFS are shown as a percentage of all patients included at the respective time points

One month after the first administration of ruxolitinib, four patients (9%) achieved CR and 13 patients (28%) PR, while four patients showed MR, 20 patients (43%) SD, and five patients (11%) PD. One patient (2%) developed clinical signs of human polyomavirus 1 (or BK virus) reactivation necessitating termination of ruxolitinib treatment and another patient was excluded from the response assessment due to an episode of intestinal aGvHD necessitating additional ISM. The ORR at 1 month was 36% (17/47) and FFS was 98% (46/47) (Fig. 1).

#### Response to ruxolitinib at 3 months

Three months after the start of ruxolitinib therapy, three patients (7%) showed CR, 10 patients PR (22%), and 10 patients MR. Six patients (13%) had SD, while five patients (11%) had PD. Eight patients (17%) received an additional ISM due to new organ involvement (including eyes, lung, skin, oral mucosa, fascia) or lack of response to ruxolitinib,

Of note, in one patient (2%) with PR, ruxolitinib had to be discontinued due to thrombocytopenia and molecular relapse of the underlying malignancy 40 days after the onset of treatment. The same patient had PD while receiving treatment with everolimus and ibrutinib. One more patient received additional ISM due to progressive cGvHD and discontinued ruxolitinib therapy later due to severe BK- and John Cunningham (JC)- virus nephropathy. Noteworthy, one of the aforementioned patients with PD initially reached a PR, but required a dose reduction of ruxolitinib after one month of treatment due to cytopenia, and subsequently had PD, with cGvHD of the skin and fascia. Despite increasing ruxolitinib back to the baseline dose, the latter patient did not respond. One patient with CR died due to pneumonia associated with coronavirus disease 2019 (COVID-19), and one patient with PR at the last follow-up had not reached the 3-month follow-up and was excluded from the ORR and FFS calculations. Therefore, the ORR was 28% (13/46) and FFS was 74% (34/46) (Fig. 1).

#### Response to ruxolitinib at 6 months

At the 6-month follow-up, three patients (7%) had CR, 12 patients (26%) PR, and seven patients (15%) MR. Five patients (11%) had PD and five patients received additional ISMs due to progression of cGvHD (*n* = 4) or severe infectious AEs (*n* = 1).

Additionally, in one patient (2%) with PR, treatment was changed to ixazomib after four months ruxolitinib due to molecular relapse of multiple myeloma and anemia.

Of note, one of the patients with PR also experienced molecular relapse after five months and continued with prednisolone monotherapy. One more patient showing SD at the last follow-up did not reach the 6-month follow-up and was excluded from the ORR and FFS calculations, resulting in an ORR of 33% (15/45) and an FFS of 58% (26/45) (Fig. 1).

#### Response to ruxolitinib at 12 months

Twelve months after the start of ruxolitinib therapy, six patients (14%) showed CR, five patients (12%) PR, two patients (5%) MR, and one patient (2%) SD, while another patient experienced PD. Three additional patients (two patients with PR and one patient with MR at last follow-up) did not reach the 12-month follow-up (total *n* = 5) and were excluded from calculations of ORR and FFS.

Six patients (14%) changed ISM due to new organ involvement, MR, or PD, while one patient changed ISM due to a local relapse of lymphoma and progressive cGvHD. Another patient, who experienced molecular relapse before the start of ruxolitinib treatment and discontinued ruxolitinib after 43 days due to cytopenia resulting in prednisolone monotherapy, now showed progressive cGvHD of the lung with need for additional ISM and increasing level of minimal residual disease. Another patient (2%) died of pneumonia, resulting in an ORR of 26% (11/42) and an FFS rate of 36% (15/42) (Fig. 1). In total, during the 12-month follow-up, two patients died due to NRM, and four patients experienced relapse of the underlying hematologic malignancy.

Taken together, ruxolitinib treatment was discontinued or another ISM was given due to lack of response for cGvHD in 18 patients and in one patient due to a new episode of aGvHD. Ruxolitinib administration was discontinued in two patients due to relapse of the underlying malignancy, due to cytopenia plus relapse of underlying malignancy in another two patients and due to cytopenia solely in one patient. Similarly, ruxolitinib treatment was discontinued due to infectious AEs in five patients. Three of the latter patients lacked response to ruxolitinib treatment, while two of them presented with CR at discontinuation. In one patient, ruxolitinib was terminated due to cytopenia in combination with an infection. Of note, in five patients responding to ruxolitinib, treatment was successfully terminated without any flare of cGvHD. In another patient with PR, ruxolitinib treatment was terminated due to relapse of multiple myeloma after 14 months. The latter patient continued with prednisolone monotherapy and did not display any flare of cGvHD within further follow-up period of three months.

Three patients that did not initially respond to ruxolitinib were re-exposed in a later episode of cGVHD but did not respond with two of them then displaying new significant cytopenia.

#### Organ grading at 3-months follow-up

Additionally, organ grading at 3-months follow-up or, if any additional ISM has been administered before, organ grading at the respective time point was assessed. Two patients, in whom new ISM has been added before 1-month follow-up, were excluded from assessment of organ grading. In terms of organ response of organs affected in at least five patients at start of ruxolitinib, skin (56%), oral mucosa (58%) and liver manifestations (83%) were more likely to respond to ruxolitinib treatment compared to eyes (11%), lung (36%) and fascia (7%) (Table 3).

**Table 3:** Organ grading at 3-months follow-up, or, in case of any additional ISM before 3-months follow-up at the respective time point (excluding two patients with new ISM before 1-month follow-up)

Type of cGvHD organ involvement	CR or PR at reported follow-up time point/nr. of patients with involvement of respective organ at ruxolitinib start (%)
Skin	18/32 (56)
Oral	14/24 (58)
Eyes	3/27 (11)
Gut	3/4 (75)
Liver	5/6 (83)
Lung	4/11 (36)
Musculoskeletal	1/15 (7)
Genital	2/2 (100)
Polyserositis	0/1 (0)
Meningoencephalitis	1/1 (100)
Polyneuropathy	0/1 (0)
Kidney involvement	0/1 (0)

#### Safety - infectious and other adverse events during ruxolitinib treatment

Infectious AEs during ruxolitinib treatment were captured within the first six months after the onset of therapy. Within this timeframe, 29 patients (60%) developed an infectious AE. 10 patients included in the analysis developed significant infectious AEs (≥ CTCAE grade III) with one patient succumbing to COVID-19 pneumonia, and another patient due to Klebsiella pneumoniae. Of note, one patient suffered from life threating CMV-colitis and one patient from ruxolitinib associated BK- and JC-virus nephropathy [18].

In total, 44 events of infectious AEs were documented within the first six months of treatment. Of those, 25 events were viral infections, with Epstein-Barr virus reactivation (*n* = 6), COVID-19 pneumonia (*n* = 5), and BK virus reactivation (*n* = 4) being the most frequent.

At onset of ruxolitinib treatment, 23 patients presented with anemia and 24 patients with thrombocytopenia of any grade (3 each with CTCAE ≥ grade III), respectively, whereas no patient had neutropenia. Within six months, 36 patients had anemia and 25 patients had thrombocytopenia of any grade (14/3 ≥ CTCAE grade III), showing a significant increase in anemia (*p* < 0.001 and *p* = 0.003, respectively), but not in thrombocytopenia. In addition, six patients had developed neutropenia, (*p* = 0.031) (Table 4).

**Table 4:** Safety within 6-months follow-up

	At start of ruxolitinib AE of any grade	Within first 6 months AE of any grade	p-value	At start of ruxolitinib SAE (CTCAE °III-IV)	Within first 6 months SAE (CTCAE °III-IV)	p-value
*Anemia, no. of pts. (%)*	23 (48)	36 (75)	< 0.001	3 (6)	14 (29)	0.003
*Thrombopenia, no. of pts. (%)*	24 (50)	25 (52)	0.999	3 (6)	3 (6)	0.999
*Neutropenia, no. of pts. (%)*	0	6 (12)	0.031	0	1 (2)	0.999
*Infectious complications – no. of pts. (%)*	-	29 (60)	-	-	10 (21)	-
*Infectious complications - Events*		44			15	
*Bacterial/fungal/unknown origin – Events* Pneumonia Sepsis Soft tissue infection/abscess Urinary tract infection Eye infection Fungal infection (candida) Bacteriemia Unknown focus		19 2 4 4 2 1 2 1 3			8 2 4 2 - - - - -	
*Viral - Events* COVID-19 pneumonia Eppstein Barr virus BK virus Influenza RSV HBV Viral gastroenteritis not specified JC virus		25 5 6 4 4 1 1 3 1			7 3 - 1 1 - - 1 1	

Abbreviations: AE = adverse event; BK virus, human polyomavirus 1; COVID-19, coronavirus disease 2019; CTCAE = Common Terminology Criteria for Adverse Events; HBV, hepatitis B virus; JC virus, John Cunningham; no. of pts.=number of patients; RSV, respiratory syncytial virus; SAE = severe AE

#### Steroid-sparing effect of ruxolitinib

**Fig. 2:**
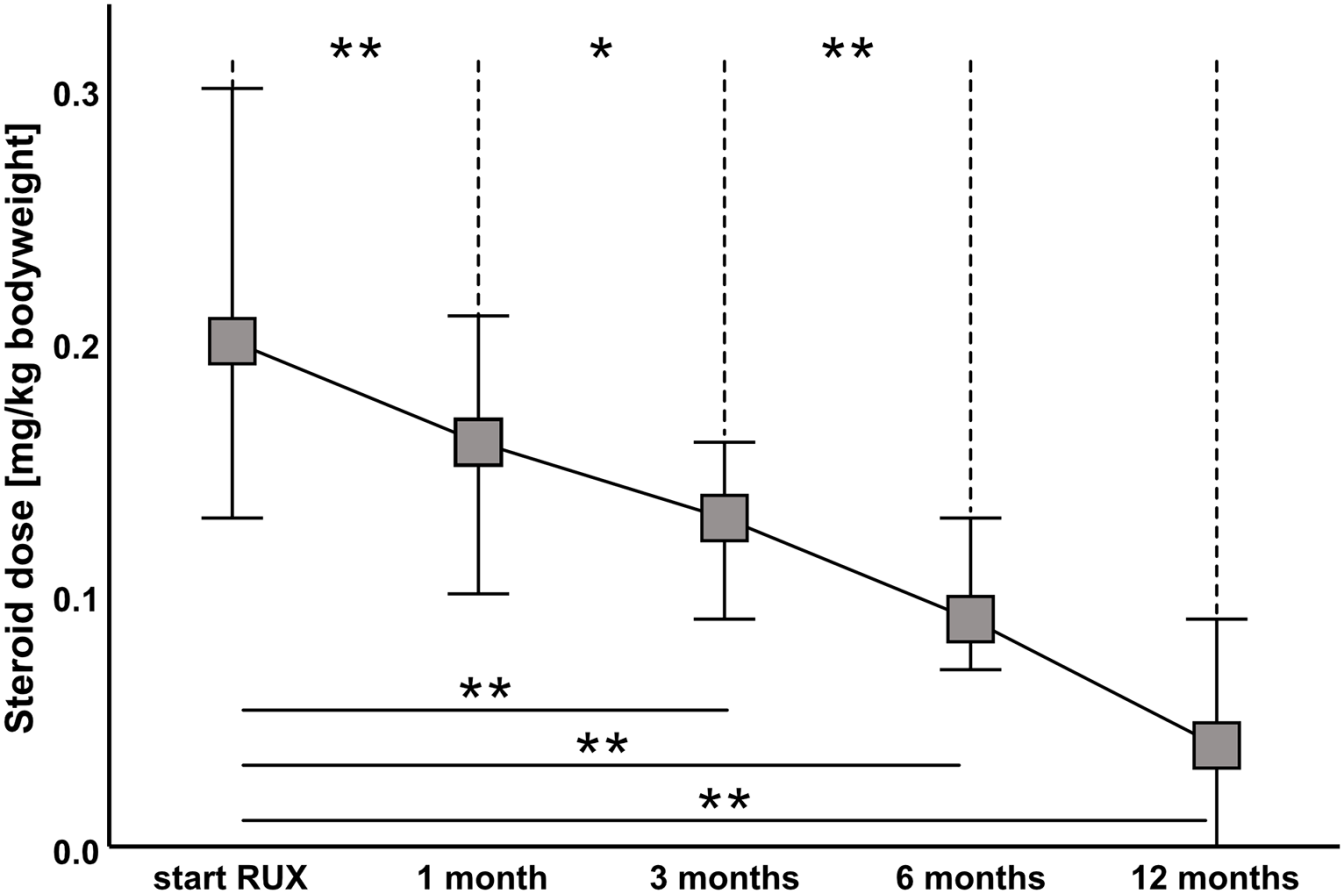
Tapering of steroid dose during follow-up. Steroid dose per kg bodyweight within a 12-month follow-up period. A significant reduction is observed. (P value 0.05 > * >0.01 > ** >0.005, treatment-induced changes are analyzed with Wilcoxon test, data are presented as median with interquartile range). A direct comparison of the steroid dose between the 6- and 12-month follow-up did not show a significant decrease, most likely as with the statistical method chosen, only those patients from the 6-month follow-up, who were still represented in the 12-month follow-up, are included in the latter calculation. RUX = ruxolitinib.

As shown in Fig. 2, the median steroid dose was constantly reduced during follow-up. After one month, the median steroid dose dropped significantly from 0.20 mg/kg (range, 0.13–0.30 mg/kg) to 0.16 mg/kg (range, 0.10–0.21 mg/kg) (*p* ≤ 0.001). At 3 months, 6 months, and 12 months after initiation of ruxolitinib, the median steroid dose was reduced to 0.13 mg/kg (range, 0.09–0.16 mg/kg; *p* ≤ 0.001), 0.09 mg/kg (range, 0.07–0.13 mg/kg; *p* ≤ 0.001) and 0.04 mg/kg (range, 0.00–0.08 mg/kg; *p* < 0.001), respectively.

## Discussion

Steroid-refractory cGvHD poses a therapeutic challenge for patients due to the fact that empiric second-line treatments show unsatisfactory outcomes [11, 19]. Since cGvHD reduces quality of life and significantly contributes to NRM, there is still a high clinical need for effective second-line treatments [20, 21]. The REACH3 trial showed a superior response rate and FFS for ruxolitinib compared with best available therapy (BAT) for the first time (ORR after 6 months: ruxolitinib 49.7% vs. BAT 25.6%; FFS: 74.9% vs. 44.5%) and ruxolitinib has since then been approved in steroid-refractory cGVHD [12, 16, 17]. In this trial, ruxolitinib was given as a second-line treatment only and the study excluded patients with pre-existing cytopenia and infectious complications. This was the rationale to assess the response rate and safety of ruxolitinib within a single-center, retrospective analysis conducted on unselected patients, including those receiving advanced treatment lines.

In our study we observed an ORR of 33% within 6 months of treatment. This response rate is lower compared with that in previously published trials [12, 22–25], in which ORRs ranged from 44 to 74%. This difference may be explained by the fact that 63% of patients in this analysis were treated with ruxolitinib after failure of second-line treatment, including patients with sclerosing manifestations. Moreover, our cohort of unselected patients treated with ruxolitinib included patients with donor lymphocyte infusion-induced cGvHD (*n* = 5), who are usually excluded from clinical trials in which cGvHD is typically treated less aggressively initially, resulting in more severe forms. Nevertheless, ORR was better than that reported in the REACH3 trial for patients that received BAT (ORR after 6 months: 25.6%).

In line with the previously published data [26], response to ruxolitinib was not associated with the severity of cGvHD, number of additional ISMs, or prior therapy lines. The univariate regression analysis did not show differences for age, sex, ruxolitinib dose at onset, azole comedication, steroid dose at onset, CTCAE platelets, and CTCAE hemoglobin at the start of ruxolitinib treatment between responder and non-responder.

A major concern in patients treated with ruxolitinib is the increased risk of cytopenia and infectious morbidity. In line with the results from the REACH3 trial, the most common adverse event was anemia, which is not surprising taking into account the mechanism of action with JAK inhibition interfering with erythropoietin receptor signaling and the reported safety profile of ruxolitinib [27, 28]. Neutropenia, another known side effect of ruxolitinib, also increased significantly with therapy. In terms of ≥ CTCAE grade III cytopenia, the number of patients with anemia, but not neutropenia, significantly increased within the first 6 months after the onset of therapy. However, both anemia and neutropenia can be well managed with supportive care, including the use of erythropoietin or G-CSF. In contrast, there was no significant increasement in thrombocytopenia. Considering that thrombocytopenia can also be due to cGvHD itself, we investigated whether there was a difference between CTCAE score at onset of therapy and during follow-up period between responder and non-responder to ruxolitinib treatment. Interestingly, within the cohort of non-responder, 61% maintained the same CTCAE score during follow-up, whereas 13% had a lower CTCAE score and 26% a higher CTCAE score compared to start of ruxolitinib treatment. In contrast, 76% of the patients responding to ruxolitinib treatment maintained the same CTCAE score, while 18% displayed a lower CTCAE score and only 6% a higher CTCAE score than before. Therefore, the lack of increase in thrombocytopenia could also be a result of an effective GvHD therapy.

The incidence of infections ≥ grade III per patient was 21% (*n* = 10), comparable to the findings from the REACH3 trial [12]. Three patients needed intensive care and two of them died during ruxolitinib treatment due to pulmonary infectious complications. Of note, one patient suffered from severe BK virus reactivation, and 4 patients in total presented with BK viremia. BK virus replication is controlled by binding of interferon-γ to its surface receptor (IFNγR), resulting in JAK/STAT pathway activation. Targeting of the IFNγR downstream kinases JAK1 and JAK2 by ruxolitinib thereby impairs the antiviral properties of IFNγ [29]. Taken together, in the present study, the safety profile was consistent with the previously reported data [12, 24, 26]. Nonetheless, these data reflect the importance of continuous surveillance of outpatients for infectious complications.

Of note, we observed a meaningful reduction in the steroid dose by a median of 20%, 35%, 55%, and 75% after 1, 3, 6, and 12 months, respectively, demonstrating a steroid-sparing activity of ruxolitinib.

In conclusion, ruxolitinib is an effective treatment option for cGvHD including efficacy in advanced treatment lines.

## Data Availability

The data that support the findings of this study are available from the corresponding author upon reasonable request.

## References

[1] Fraser CJ, Bhatia S, Ness K, Carter A, Francisco L, Arora M, Parker P, Forman S, Weisdorf D, Gurney JG, Baker KS (2006) Impact of chronic graft-versus-host disease on the health status of hematopoietic cell transplantation survivors: a report from the bone marrow transplant Survivor Study. Blood 108(8):2867–2873. 10.1182/blood-2006-02-003954 16788100 10.1182/blood-2006-02-003954 PMC1895593

[2] Pidala J, Kurland B, Chai X, Majhail N, Weisdorf DJ, Pavletic S, Cutler C, Jacobsohn D, Palmer J, Arai S, Jagasia M, Lee SJ (2011) Patient-reported quality of life is associated with severity of chronic graft-versus-host disease as measured by NIH criteria: report on baseline data from the chronic GVHD Consortium. Blood 117(17):4651–4657. 10.1182/blood-2010-11-319509 21355084 10.1182/blood-2010-11-319509 PMC3099579

[3] Arora M, Cutler CS, Jagasia MH, Pidala J, Chai X, Martin PJ, Flowers ME, Inamoto Y, Chen GL, Wood WA, Khera N, Palmer J, Duong H, Arai S, Mayer S, Pusic I, Lee SJ (2016) Late Acute and Chronic Graft-versus-host disease after allogeneic hematopoietic cell transplantation. Biol Blood Marrow Transpl 22(3):449–455. 10.1016/j.bbmt.2015.10.018 10.1016/j.bbmt.2015.10.018 PMC4787270 26541363

[4] Bolaños-Meade J, Hamadani M, Wu J, Al Malki MM, Martens MJ, Runaas L, Elmariah H, Rezvani AR, Gooptu M, Larkin KT, Shaffer BC, El Jurdi N, Loren AW, Solh M, Hall AC, Alousi AM, Jamy OH, Perales MA, Yao JM, Applegate K, Bhatt AS, Kean LS, Efebera YA, Reshef R, Clark W, DiFronzo NL, Leifer E, Horowitz MM, Jones RJ, Holtan SG, BMT CTN 1703 Investigators (2023) N Engl J Med 388(25):2338–2348. 10.1056/NEJMoa2215943. Post-Transplantation Cyclophosphamide-Based Graft-versus-Host Disease Prophylaxis 37342922 10.1056/NEJMoa2215943 PMC10575613

[5] Kröger N, Solano C, Wolschke C, Bandini G, Patriarca F, Pini M, Nagler A, Selleri C, Risitano A, Messina G, Bethge W, Pérez de Oteiza J, Duarte R, Carella AM, Cimminiello M, Guidi S, Finke J, Mordini N, Ferra C, Sierra J, Russo D, Petrini M, Milone G, Benedetti F, Heinzelmann M, Pastore D, Jurado M, Terruzzi E, Narni F, Völp A, Ayuk F, Ruutu T, Bonifazi F (2016) Antilymphocyte Globulin for Prevention of Chronic Graft-versus-host disease. N Engl J Med 374(1):43–53. 10.1056/NEJMoa1506002 26735993 10.1056/NEJMoa1506002

[6] Zeiser R, Longo DL, Blazar BR (2017) Pathophysiology of chronic graft-versus-host disease and therapeutic targets. N Engl J Med 377(26):2565–2579. 10.1056/NEJMra1703472 29281578 10.1056/NEJMra1703472

[7] MacDonald KPA, Hill GR, Blazar BR (2017) Chronic graft-versus-host disease: biological insights from preclinical and clinical studies. Blood 129(1):13–21. 10.1182/blood-2016-06-686618 27821504 10.1182/blood-2016-06-686618 PMC5216261

[8] Wolff D, Lawitschka A (2019) Chronic Graft-Versus-Host Disease. In: Carreras E, Dufour C, Mohty M, Kroger N (eds) The EBMT Handbook: Hematopoietic Stem Cell Transplantation and Cellular Therapies. 7th edn., Cham (CH), pp 331–345. 10.1007/978-3-030-02278-5_44

[9] Wolff D, Gerbitz A, Ayuk F, Kiani A, Hildebrandt GC, Vogelsang GB, Elad S, Lawitschka A, Socie G, Pavletic SZ, Holler E, Greinix H (2010) Consensus Conference on Clinical Practice in Chronic Graft-versus-Host Disease (GVHD): First-Line and Topical Treatment of Chronic GVHD. Biology of Blood and Marrow Transplantation 16 (12):1611–1628. 10.1016/j.bbmt.2010.06.015 10.1016/j.bbmt.2010.06.015 20601036

[10] Jagasia MH, Greinix HT, Arora M, Williams KM, Wolff D, Cowen EW, Palmer J, Weisdorf D, Treister NS, Cheng GS, Kerr H, Stratton P, Duarte RF, McDonald GB, Inamoto Y, Vigorito A, Arai S, Datiles MB, Jacobsohn D, Heller T, Kitko CL, Mitchell SA, Martin PJ, Shulman H, Wu RS, Cutler CS, Vogelsang GB, Lee SJ, Pavletic SZ, Flowers ME (2015) National Institutes of Health Consensus Development Project on Criteria for clinical trials in chronic graft-versus-host disease: I. The 2014 diagnosis and Staging Working Group report. Biol Blood Marrow Transpl 21(3):389–401e381. 10.1016/j.bbmt.2014.12.001 10.1016/j.bbmt.2014.12.001 PMC4329079 25529383

[11] Axt L, Naumann A, Toennies J, Haen SP, Vogel W, Schneidawind D, Wirths S, Moehle R, Faul C, Kanz L, Axt S, Bethge WA (2019) Retrospective single center analysis of outcome, risk factors and therapy in steroid refractory graft-versus-host disease after allogeneic hematopoietic cell transplantation. Bone Marrow Transplant 54(11):1805–1814. 10.1038/s41409-019-0544-y 31089279 10.1038/s41409-019-0544-y

[12] Zeiser R, Polverelli N, Ram R, Hashmi SK, Chakraverty R, Middeke JM, Musso M, Giebel S, Uzay A, Langmuir P, Hollaender N, Gowda M, Stefanelli T, Lee SJ, Teshima T, Locatelli F (2021) Ruxolitinib for glucocorticoid-refractory chronic graft-versus-host disease. N Engl J Med 385(3):228–238. 10.1056/NEJMoa2033122 34260836 10.1056/NEJMoa2033122

[13] Elli EM, Baratè C, Mendicino F, Palandri F, Palumbo GA (2019) Mechanisms underlying the anti-inflammatory and immunosuppressive activity of Ruxolitinib. Front Oncol 9. 10.3389/fonc.2019.01186 10.3389/fonc.2019.01186 PMC6854013 31788449

[14] Heine A, Held SAE, Daecke SN, Wallner S, Yajnanarayana SP, Kurts C, Wolf D, Brossart P (2013) The JAK-inhibitor ruxolitinib impairs dendritic cell function in vitro and in vivo. Blood 122(7):1192–1202. 10.1182/blood-2013-03-484642 23770777 10.1182/blood-2013-03-484642

[15] Przepiorka D, Luo L, Subramaniam S, Qiu J, Gudi R, Cunningham LC, Nie L, Leong R, Ma L, Sheth C, Deisseroth A, Goldberg KB, Blumenthal GM, Pazdur R (2020) FDA approval Summary: Ruxolitinib for treatment of steroid-refractory Acute graft-versus-host disease. Oncologist 25(2):e328–e334. 10.1634/theoncologist.2019-0627 32043777 10.1634/theoncologist.2019-0627 PMC7011641

[16] Wolf A, Masow J, Billings M (2022) Novartis receives European Commission approval for Jakavi® to be the first post-steroid treatment for acute and chronic graft-versus-host disease. Novartis.com

[17] Martini DJ, Chen Y-B, DeFilipp Z (2022) Recent FDA approvals in the treatment of graft-versus-host disease. Oncologist 27(8):685–693. 10.1093/oncolo/oyac076 35443042 10.1093/oncolo/oyac076 PMC9355804

[18] Fante MA, Holler E, Schmidt B, Wolff D, Ehrl Y, Plentz A (2018) Persistent polyomavirus-associated nephropathy in a patient with GvHD and treatment with the JAK1/2 inhibitor ruxolitinib. Bone Marrow Transplant 54(5):762–764. 10.1038/s41409-018-0370-7 30349038 10.1038/s41409-018-0370-7

[19] Wolff D, Fatobene G, Rocha V, Kröger N, Flowers ME (2021) Steroid-refractory chronic graft-versus-host disease: treatment options and patient management. Bone Marrow Transplant 56(9):2079–2087. 10.1038/s41409-021-01389-5 34218265 10.1038/s41409-021-01389-5 PMC8410585

[20] Garnett C, Apperley JF, Pavlů J (2013) Treatment and management of graft-versus-host disease: improving response and survival. Therapeutic Adv Hematol 4(6):366–378. 10.1177/2040620713489842 10.1177/2040620713489842 PMC3854558 24319572

[21] Flowers MED, Martin PJ (2015) How we treat chronic graft-versus-host disease. Blood 125(4):606–615. 10.1182/blood-2014-08-551994 25398933 10.1182/blood-2014-08-551994 PMC4304105

[22] Wang D, Liu Y, Lai X, Chen J, Cheng Q, Ma X, Lin Z, Wu D, Xu Y (2021) Efficiency and toxicity of Ruxolitinib as a salvage treatment for steroid-refractory chronic graft-versus-host disease. Front Immunol 12. 10.3389/fimmu.2021.673636 10.3389/fimmu.2021.673636 PMC8278571 34276662

[23] Modi B, Hernandez-Henderson M, Yang D, Klein J, Dadwal S, Kopp E, Huelsman K, Mokhtari S, Ali H, Malki MMA, Spielberger R, Salhotra A, Zain J, Cotliar J, Parker P, Forman S, Nakamura R (2019) Ruxolitinib as Salvage Therapy for Chronic Graft-versus-host disease. Biol Blood Marrow Transplant 25(2):265–269. 10.1016/j.bbmt.2018.09.003 30201397 10.1016/j.bbmt.2018.09.003

[24] Wu H, Shi J, Luo Y, Tan Y, Zhang M, Lai X, Yu J, Liu L, Fu H, Huang H, Zhao Y (2021) Evaluation of Ruxolitinib for Steroid-Refractory Chronic Graft-vs-host disease after allogeneic hematopoietic stem cell transplantation. JAMA Netw Open 4(1):e2034750. 10.1001/jamanetworkopen.2020.34750 33502484 10.1001/jamanetworkopen.2020.34750 PMC7841467

[25] Xue E, Lorentino F, Pavesi F, Assanelli A, Peccatori J, Bernardi M, Corti C, Ciceri F, Lupo Stanghellini MT (2021) Ruxolitinib for chronic steroid-refractory graft versus host disease: a single center experience. Leuk Res 109. 10.1016/j.leukres.2021.106642 10.1016/j.leukres.2021.106642 34157510

[26] Redondo S, Esquirol A, Novelli S, Caballero AC, Garrido A, Oñate G, López J, Moreno C, Saavedra S-D, Granell M, Briones J, Sierra J, Martino R, García-Cadenas I (2022) Efficacy and safety of Ruxolitinib in Steroid-Refractory/Dependent Chronic Graft-versus-host disease: real-World data and challenges. Transplantation Cell Therapy 28(1):43. e41-43.e45 10.1016/j.jtct.2021.10.015 34757054

[27] Verstovsek S, Mesa RA, Gotlib J, Levy RS, Gupta V, DiPersio JF, Catalano JV, Deininger M, Miller C, Silver RT, Talpaz M, Winton EF, Harvey JH, Arcasoy MO, Hexner E, Lyons RM, Paquette R, Raza A, Vaddi K, Erickson-Viitanen S, Koumenis IL, Sun W, Sandor V, Kantarjian HM (2012) A Double-Blind, placebo-controlled trial of Ruxolitinib for Myelofibrosis. N Engl J Med 366(9):799–807. 10.1056/NEJMoa1110557 22375971 10.1056/NEJMoa1110557 PMC4822164

[28] Harrison C, Kiladjian J-J, Al-Ali HK, Gisslinger H, Waltzman R, Stalbovskaya V, McQuitty M, Hunter DS, Levy R, Knoops L, Cervantes F, Vannucchi AM, Barbui T, Barosi G (2012) JAK inhibition with Ruxolitinib versus best available therapy for myelofibrosis. N Engl J Med 366(9):787–798. 10.1056/NEJMoa1110556 22375970 10.1056/NEJMoa1110556

[29] Abend JR, Low JA, Imperiale MJ (2007) Inhibitory effect of Gamma Interferon on BK Virus Gene expression and replication. J Virol 81(1):272–279. 10.1128/jvi.01571-06 17035315 10.1128/jvi.01571-06 PMC1797268

